# Clinical Features and Treatment Outcomes among Children with Stevens-Johnson Syndrome and Toxic Epidermal Necrolysis: A 20-Year Study in a Tertiary Referral Hospital

**DOI:** 10.1155/2018/3061084

**Published:** 2018-05-07

**Authors:** Susheera Chatproedprai, Vanvara Wutticharoenwong, Therdpong Tempark, Siriwan Wananukul

**Affiliations:** Department of Pediatrics, Faculty of Medicine, Chulalongkorn University and King Chulalongkorn Memorial Hospital, Bangkok 10330, Thailand

## Abstract

**Aim:**

To determine the probable causative factors, clinical features, and treatment outcomes of Stevens-Johnson syndrome (SJS), toxic epidermal necrolysis (TEN), and SJS-TEN overlap in children.

**Methods:**

A 20-year database review of all children diagnosed with SJS/TEN/SJS-TEN overlap at the King Chulalongkorn Memorial Hospital, Thailand.

**Results:**

36 patients (M : F, 16 : 20) with the mean age of 9.2 ± 4.0 years were identified. There were 20 cases of SJS, 4 cases of SJS-TEN overlap, and 12 cases of TEN. Drugs were the leading cause for the diseases (72.3%); antiepileptics were the most common culprits (36.1%). Cutaneous morphology at presentation was morbilliform rash (83.3%), blister (38.9%), targetoid lesions (25.0%), and purpuric macules (2.8%). Oral mucosa (97.2%) and eye (83.3%) were the 2 most common mucosal involvements. Majority of the cases (77.8%) were treated with systemic corticosteroids, intravenous immunoglobulin, or both. Treatment outcomes between those who received systemic therapy and those who received only supportive care were comparable. Skin and eye were the principal sites of short-term and long-term complications.

**Conclusions:**

SJS/TEN are not common but are serious diseases which lead to significant morbidities in children. Early withdrawal of suspicious causes and meticulous supportive care are very important. This study found that the systemic therapy was not superior to supportive care because the treatment outcomes for both groups were comparable.

## 1. Introduction

Stevens-Johnson syndrome (SJS), toxic epidermal necrolysis (TEN), and SJS-TEN overlap are rare but serious diseases. They are considered to be the same spectrum of diseases, defined by the area of epidermal detachment. SJS is the mildest form affecting <10% of the body surface area (BSA). TEN is the most severe disease affecting >30% of the BSA. Total BSA involvement of 10–30% is defined as SJS-TEN overlap. The overall incidence of SJS and TEN was 0.4–6 cases per 1,000,000 persons [1, 2].

The optimal treatment for SJS/TEN is inconclusive. Meticulous skin care, hydration, pain control, early identification, and discontinuation of the probable culprit drug as well as an early admission to a specialised unit are the most important things in controlling the disease. Systemic corticosteroids (SCS) and/or intravenous immunoglobulin (IVIG) were proposed systemic treatments but their efficacy remains debatable. The mortality rate is high for SJS and significantly higher for TEN. The reported mortality rate in the Thai population was 7–50% [3] which is quite higher relative to other countries [4, 5].

This study assessed the probable causative factors, clinical features, and treatment outcomes in SJS/TEN/SJS-TEN overlap pediatric patients in a tertiary referral hospital.

## 2. Patients and Methods

This retrospective study was approved by the Institutional Review Board (IRB), Faculty of Medicine, Chulalongkorn University, and adheres to the provisions outlined in the Declaration of Helsinki (IRB number 316/55).

### 2.1. Participants

The database for all pediatric inpatients (<18 years old) diagnosed with SJS, TEN, or SJS-TEN, overlap by pediatric dermatologists and admitted to the King Chulalongkorn Memorial Hospital, Bangkok, Thailand, from January 1997 to December 2016 was retrospectively reviewed.

The following data were recorded: (1) history such as demographic data, comorbidity, past medical and allergic history, previous exposure to the inciting drug, and history of concurrent infection, (2) clinical features such as prodromal symptoms, cutaneous lesions (BSA involvement, pattern, and distribution of lesions), number and area of mucosal involvement, (3) treatment such as supportive care (wound care, hydration, and pain control) and/or systemic treatment (SCS and/or IVIG), and (4) outcomes such as duration of hospital stay, short-term complication, and long-term sequel. Long-term sequel was defined as observed end-organ failure after resolution of SJS/TEN, or the onset of another disease during the acute stage which did not resolve at least one month after the resolution of SJS/TEN [6]. Causative drugs were determined by considering the timeline of drug administration and disease occurrence. For the first time exposure, the interval between drug administration and the onset of symptoms should be within a week to a month and less than 2 weeks in the patients with history of re-exposure. In addition, the causative drugs were also identified by the result from the patch test if it was performed.

### 2.2. Statistical Analysis

Categorical data are presented as number and percentage. Continuous data are expressed as mean and standard deviation (SD). The statistical analysis was performed using Chi-Square test for proportion and Mann–Whitney *U* test for continuous data. All statistical analyses were done using the SPSS version 20 (IBM Corp, New York, NY, USA). *P* value < 0.05 was considered statistically significant.

## 3. Results

A total of 36 patients (male *n* = 16, 44.4%) with the mean age (SD) of 9.2 (4.0) years were identified for the study. The age ranged from 1 year 5 months to 15 years 10 months. There were 20 cases of SJS, 4 cases of SJS-TEN overlap, and 12 cases of TEN. The sample size for SJS-TEN overlap patients was small so the authors combined the patients from the SJS-TEN overlap group with the patients from the TEN group and reclassified them as overlap-TEN group.

### 3.1. Demographic Data

Fifteen patients (41.6%) were previously healthy. The remaining 21 cases had underlying diseases. Twelve (33.3%) of them had neurological diseases, followed by human immunodeficiency virus (*n* = 3, 8.3%), end-stage renal disease (*n* = 2, 5.6%), Wilson disease (*n* = 2, 5.6%), systemic lupus erythematosus (*n* = 1, 2.8%), and cyanotic heart disease (*n* = 1, 2.8%) (Table 1).

### 3.2. Causative Factors

For majority of the patients, the cause for the disease was prescription drugs (*n* = 26, 72.3%). The leading culprit was the antiepileptic drugs (*n* = 13, 36.1%), followed by antibiotics (*n* = 9, 25.0%). In the SJS group, antibiotics (35.0%) were the leading cause for the disease. In the overlap-TEN group, the most common causes for the disease were antiepileptics (50.0%) followed by antibiotics (12.5%).

Almost all patients were investigated to exclude the possible infectious causative factors including* Mycoplasma pneumoniae* and/or herpes simplex virus and/or Epstein-Barr virus. The results were all negative except for 3 cases. For 1 case (5%) from the SJS group, infection was the possible cause for the disease but the organism could not be identified. In three cases (18.8%) from the overlap-TEN group, infections were the causes of the disease. Two of them had* Mycoplasma pneumoniae* infection and 1 case had Epstein-Barr virus infection. All of them had no previous history of any drug exposure prior to the lesions. On the other hand, of 36 cases, we could not identify causes of the disease in 6 cases (16.6%) (Table 1).

Concerning the timeline for considering a drug as suspicious, duration from drug administration to disease occurrence was 12.6 ± 3.9 days (7–21 days) for patients with history of first-time exposure and 3.3 ± 3.8 days (1–10 days) for those with previous exposure (*P* < 0.001). However, the duration was comparable between SJS group (8.6 ± 6.6 days) and overlap-TEN group (9.4 ± 5.0 days).

### 3.3. Clinical Features

Seventy-five percent of SJS group and 68.8% of overlap-TEN group had prodromal symptoms. Fever was the main complaint, ranging from 1 to 7 days with the mean duration (SD) of 1.3 (1.5) days, followed by stinging eyes and sore throat. The most common cutaneous morphology at the presentation was morbilliform rash, including maculopapular rash and exanthematous rash (95.0% in SJS; 68.8% in overlap-TEN); there were significant differences between the SJS and overlap-TEN groups for morbilliform rash (*P* = 0.036). Purpura was found in only 1 case (5.0%) from the SJS group. Five patients (25%) from the SJS group and 4 cases (25%) from the overlap-TEN had both blister and morbilliform rash.

The 2 most common areas of mucosal involvement in this study were oral (*n* = 35, 97.2%) and eye (*n* = 30, 83.3%) but this was not statistically significant for both groups (Table 2). The number of sites with mucosal involvement varied between 2, 3, and 4 sites. For SJS cases, 45.0% (*n* = 9) involved 2 mucosal sites, 40.0% (*n* = 8) involved 3 mucosal sites, and 15.0% (*n* = 3) involved 4 mucosal sites. For overlap-TEN cases, 50.0% (*n* = 8) involved 2 mucosal sites, 31.2% (*n* = 5) involved 3 mucosal sites, and 18.8% (*n* = 3) involved 4 mucosal sites.

Associated abnormalities and visceral organs involvement in this study were predominantly associated with GI abnormalities (*n* = 18, 50.0%), especially transaminitis (*n* = 18, 50.0%) and electrolyte abnormalities (*n* = 16, 44.4%) but this was not significant between both groups (Table 2).

### 3.4. Treatment

All cases were treated with multidisciplinary team, meticulous wound care with or without dressings (Acticoat™, Biobrane™), hydration, pain control, and isolation at either intensive care unit or a specialised unit as the supportive care. Majority of cases in the SJS group (*n* = 16, 80.0%) and overlap-TEN group (*n* = 12, 75.0%) were treated with systemic treatment (systemic corticosteroids (SCS) at the dose equivalent to prednisolone 1–4 mg/kg/day, intravenous immunoglobulin (IVIG) at the total dose 2–7 mg/kg, or both). All 16 cases from the SJS group, who received systemic treatment, were treated with SCS. The duration of SCS treatment including tapering period varied from 7 to 60 days with the mean (SD) of 22.9 (14.5) days.

In regard to the overlap-TEN group who received systemic treatment (*n* = 12), 8 cases (66.7%) were treated with SCS alone, 1 case (8.3%) was treated with IVIG only at the dose of 1 mg/kg/day for 4 days, and 3 cases (25.0%) were treated with both SCS and IVIG. Of these 3 cases treated with both SCS and IVIG, 1 case with severe epidermal detachment (>90% BSA) received IVIG 1 mg/kg/day for 3 days and SCS at the dose equivalent to prednisolone 4 mg/kg/day but the degree of skin detachment still progressed so additional IVIG 2 mg/kg/day for 2 days was prescribed. The duration of SCS treatment in this group ranged from 4 to 69 days with the mean (SD) of 39.3 (21.0) days. There was no significant difference between the duration of SCS treatment between the SJS group and the overlap-TEN group (*P* = 0.171).

### 3.5. Treatment Outcomes

The duration of hospital stay was significantly different between the SJS (7.1 ± 4.1 days) and overlap-TEN groups (17.7 ± 13.1 days) (*P* < 0.001). Also, the overall short-term eye complication was significantly different between the SJS (*n* = 10, 50.0%) and overlap-TEN groups (*n* = 14, 87.5%) (*P* = 0.018). In addition, skin change (dyspigmentation), nail change, and vaginal adhesion were significantly different between the SJS and overlap-TEN groups. Other outcomes such as prevalence of superinfection, pneumonia, pancreatitis, and adrenal insufficiency were comparable between the SJS and overlap-TEN groups (Table 3). No case of the SJS group had pancreatitis or adrenal insufficiency where the overlap-TEN group had 1 case (6.2%, *P* = 0.257) of each.

The mean duration of follow-up was 13.1 months (SD 19.9, 1–80 months). In regard to long-term skin sequel, dyspigmentation was the most common finding in the SJS (*n* = 4, 20.0%) and overlap-TEN (*n* = 14, 87.5%) groups (*P* < 0.001). One case (5.0%) from the SJS group had xerosis and 1 case (6.2%) from the overlap-TEN group had nail loss. For long-term eye sequel, dry eye was the single long-term complication found in the SJS group (*n* = 4, 20.0%), while, in the overlap-TEN group, dry eye was the most common long-term sequel (*n* = 6, 37.5%), followed by corneal scar (*n* = 4, 25.0%), keratopathy (*n* = 3, 18.8%), and subconjunctival fibrosis (*n* = 1, 6.2%). Transaminitis was the long-term GI problem found in the SJS group (*n* = 2, 10.0%) and overlap-TEN group (*n* = 6, 37.4%) (Table 4). There was no deceased SJS/TEN case in this study. Recurrence occurred in only 1 case from the SJS group (5.0%).

Comparing between those receiving only supportive care (*n* = 8, 22.2%) and those receiving systemic treatment (*n* = 28, 77.8%), mean (SD) of duration of hospital stay was 9.9 (7.4) and 12.3 (11.4) (*P* = 0.593). Overall short-term and long-term complications were comparable between both groups without significant differences (Table 5).

Comparisons of clinical features, laboratory findings, and treatment outcomes between children in this study and adults from previous studies were shown in Table 6.

## 4. Discussion

This study confirms the rarity of SJS/TEN cases. For our institution, a main tertiary referral hospital in Thailand, the prevalence was 1.8 cases/year. This finding is similar to other previous studies; there was a slightly higher risk for girls and a higher number of chronic health conditions associated with SJS cases [20, 21] in contrast to overlap-TEN patients. Drugs were the leading causative factors for both groups. These results are consistent with prior findings [22–24]. Antiepileptics were the most common culprit drugs for the overlap-TEN group in this study. It is thought that a reactive drug metabolite exerts a direct effect on the keratinocytes [25]. CD8+ T cells, stimulated by the causative drugs or drug metabolites, mediate keratinocyte apoptosis by at least 3 different pathways: (1) Fas/Fas ligand interaction, (2) cytotoxic T-cell, and (3) natural killer- (NK-) cell damage via perforin/granzyme B/granulysin, and tumor necrosis factor-*α* (TNF-*α*) [26, 27]. In this study, 2 cases with TEN had mycoplasma infection without the previous history of any drug exposure whereas, in other studies, only SJS cases had mycoplasma infection [23]. We did not assume that these 2 cases had mycoplasma induced rash and mucositis [28] because both of them had severe clinical courses which needed 22 and 55 days of admission and both of them encountered long-term eye sequel. There was no report of herpes simplex virus (HSV) infection in this study which contradicts previous reports that there was 9.0–19.7% of HSV infection among SJS patients [24, 29].

The most common cutaneous findings at the presentation in this study were morbilliform rash, defined as maculopapular rash or exanthematous rash, but excluding atypical targetoid macules or purpuric macules. One-fourth of cases from the SJS group and 56.3% cases (*n* = 9) from the overlap-TEN group had blisters. The difference in the prevalence of blisters in overlap-TEN group was 2.25 times more common than in SJS group but it did not reach statistical significance. The combination of SJS-TEN overlap cases with TEN cases in this study may explain the skewness of the trend. Mucosal involvement was predominant at the oral mucosa (95.0-100.0%), followed by eye (81.2–85.0%), genitalia (68.6–75.0%), and anus (15.0–18.8%). Lesions at more than 2 sites were 50.0–55.0%. The prevalence of ocular involvement, genital involvement, and lesions at more than 2 sites was slightly higher than the previous data [7, 23].

The prevalence of mucosal involvement was comparable between the SJS and over-TEN groups. However, its severity of involvement leading to either short-term complication or long-term sequel was statistically significant in overlap-TEN groups, especially for the eye (*P* = 0.018) and genital involvement (*P* = 0.043).

No case with encephalopathy was observed in the present study in contrast to Yamane's study which found 3.8% in SJS and 14.3% in TEN cases [7].

For treatments, systemic corticosteroids were mainly used in both groups. SCS treatment can decrease the percentage of perforin-positive CD8+ T lymphocytes [30] and decrease excessive immune response [7]. SCS has been suggested to be a valid treatment [31, 32] for the disease but the result of this study did not support the efficacy of SCS treatment over the supportive care alone. The duration of hospital stay, short-term and long-term sequel, and recurrence were comparable between systemic treatment group and the supportive care alone group. Although the prevalence of long-term eye sequel in cases with supportive care alone (62.5%) was 2.18 times higher than in the systemic treatment (28.6%) group, this was not statistically significant (*P* = 0.302). There was no deceased case in this study.

As for the IVIG therapy, only 1 patient in this study was treated with IVIG alone. Additional 3 cases were treated with both IVIG and SCS. Because few were treated with IVIG, therefore we cannot assess the treatment outcome of IVIG to be positive or negative. However, there were studies documenting the favourable outcome of IVIG either alone or combined with SCS in slowing the disease progression among SJS/TEN patients [23, 33, 34].

Ophthalmic complications were found in SJS/TEN with the incidence ranging from 20.0–81.0% [4, 6, 35–37]. In this study, short-term complications were seen in 66.7% of cases and long-term complications were seen in 36.1% of cases. The incidence of these complications was significantly higher in overlap-TEN group than in the SJS group for both short-term (*P* = 0.018) and long-term (*P* = 0.042) complications. In addition, this trend was significantly observed in skin dyspigmentation (*P* = 0.001), nail change (*P* = 0.018), and vaginal adhesion (*P* = 0.043).

Other complications were rare but were present in this study. A case with TEN had adrenal insufficiency and another case with SJS-TEN overlap had pancreatitis. Both of them were previously healthy and had no prior medical exposure to anything that can cause the complication.* Mycoplasma pneumoniae* was presumed to be the cause for the case with adrenal insufficiency by positive IgM serology to* Mycoplasma pneumoniae*. Epstein-Barr virus was the cause for the case with pancreatitis by positive IgM serology to viral capsid antigen. It has been reported that adrenal insufficiency and tuberculosis in an adult were associated with SJS [18]. To our knowledge, there has been no report of TEN associated with adrenal insufficiency in children. In regard to the pancreatitis, there have been only 2 reports associated with TEN and SJS in children [38, 39] and few reports in adults [15–17].

Comparing to adults' data from previous studies [6–14, 19], children tend to have higher frequency of mucosal involvement in all areas including short-term dyspigmentation. However, renal abnormalities were less frequent and there were no lung abnormalities, encephalopathy, or mortality in this study.

The limitation in the present study was that the data was from a single referral centre. Another limitation was its retrospective design. Therefore, we cannot identify the causative factors for all patients. In addition, there were few cases that were treated with IVIG so that we cannot make any assumption toward its treatment outcome. However, it is difficult and unethical to perform a randomized controlled trial for these diseases.

## 5. Conclusion

SJS/TEN are serious diseases even though they are rare diseases. Compared to the SJS group, the overlap-TEN group had more significant morbidities of the skin, eye, and genital organs. Early and prompt recognition, early withdrawal of suspicious causative factors, meticulous supportive care, and an early admission to a specialised unit are the most essential parts of managing these patients. In this study, the treatment outcomes were comparable between systemic treatment and supportive care only. This indicated that systemic treatment was not superior to supportive care only.

## Figures and Tables

**Table 1:** Demographics of patients with Stevens-Johnson syndrome and overlap-toxic epidermal necrolysis.

	SJS, *N *= 20	Overlap-TEN, *N *= 16	Overall, *N *= 36	*P* value
*Male* (%)	8 (40.0)	8 (50.0)	16 (44.4)	0.549

*Age* (yr) (mean ± SD)	8.6 ± 4.2	9.9 ± 3.6	9.2 ± 4.0	0.373

*Underlying diseases*, *N* (%)				0.623
None	7 (35.0)	8 (50.0)	15 (41.6)	
Neurological diseases	6 (30.0)	6 (37.5)	12 (33.3)	
(seizure, MELAS, GBS)				
HIV	2 (10.0)	1 (6.2)	3 (8.3)	
ESRD	1 (5.0)	1 (6.2)	2 (5.6)	
Wilson disease	2 (10.0)	0	2 (5.6)	
SLE	1 (5.0)	0	1 (2.8)	
Cyanotic heart disease	1 (5.0)	0	1 (2.8)	

*Probable causative factors* (%)				0.185
Unknown	4 (20.0)	2 (12.5)	6 (16.6)	
Infection	1 (5.0)	3 (18.8)	4 (11.1)	
Drug	15 (75.0)	11 (68.7)	26 (72.3)	
Antiepileptics	5 (25.0)	8 (50.0)	13 (36.1)	
Antibiotics	7 (35.0)	2 (12.5)	9 (25.0)	
D-penicillamine	2 (10.0)	0	2 (5.6)	
Antivirus	1 (5.0)	1 (6.2)	1 (2.8)	
NSAIDs	0	0	1 (2.8)	

MELAS, mitochondrial myopathy, encephalopathy, lactic acidosis, and stroke; GBS, Guillain-Barre syndrome; HIV, human immunodeficiency virus; ESRD, end-stage renal disease; SLE, systemic lupus erythematosus.

**Table 2:** Clinical features of patients with Stevens-Johnson syndrome and overlap-toxic epidermal necrolysis.

	SJS, *N *= 20	Overlap-TEN, *N *= 16	Overall, *N *= 36	*P* value^*∗*^
*Prodromal symptoms*, *N* (%)	15 (75.0)	11 (68.8)	26 (72.2)	0.677
Fever	14 (70.0)	11 (68.8)	25 (69.4)	0.352
Stinging eye	4 (20.0)	4 (25.0)	8 (22.2)	0.477
Sore throat	5 (25.0)	1 (6.2)	6 (16.7)	0.274

*Cutaneous findings at the presentation*, *N* (%)				
Morbilliform rash	19 (95.0)	11 (68.8)	30 (83.3)	**0.036**
Blister	5 (25.0)	9 (56.3)	14 (38.9)	0.056
Targetoid lesions	6 (30.0)	3 (18.8)	9 (25.0)	0.439
Purpuric macules	1 (5.0)	0	1 (2.8)	0.364

*Mucosal involvement*, *N* (%)				
Oral	19 (95.0)	16 (100.0)	35 (97.2)	0.364
Eye	17 (85.0)	13 (81.2)	30 (83.3)	0.764
Genital	15 (75.0)	11 (68.8)	26 (72.2)	0.677
Anus	3 (15.0)	3 (18.8)	6 (16.7)	0.764

*Associated abnormalities*				
GI abnormalities	9 (45.0)	9 (56.3)	18 (50.0)	0.502
Transaminitis	9 (45.0)	9 (56.3)	18 (50.0)	0.645
Direct hyperbilirubinemia	3 (15.0)	0	3 (8.3)	0.106
Electrolyte abnormalities	8 (40.0)	8 (50.0)	13 (44.4)	0.568
Hyponatremia	5 (25.0)	3 (18.8)	8 (22.2)	0.654
Hypokalemia	3 (15.0)	4 (25.0)	7 (19.4)	0.451
Hypocalcemia, hypophosphatemia	0	1 (6.2)	1 (2.8)	0.257
Renal abnormalities	1 (5.0)	1 (6.2)	2 (5.6)	0.359
Rising creatinine	1 (5.0)	0	1 (2.8)	0.364
Rising creatinine and hematuria	0	1 (6.2)	1 (2.8)	0.257

^*∗*^Significant values are shown in bold.

**Table 3:** Treatment and treatment outcomes of patients with Stevens-Johnson syndrome and overlap-toxic epidermal necrolysis.

	SJS,* N *= 20	Overlap-TEN, *N *= 16	Overall, *N *= 36	*P* value^*∗*^
*Treatment*, *N* (%)				
Specific treatment	16 (80.0)	75.0	77.8	0.720
Supportive treatment only	4 (20.00)	25.0	22.2	0.720

*Duration of hospital stay* (d) (mean ± SD)	7.1 ± 4.1	17.7 ± 13.1	11.8 ± 10.6	**<0.001**

*Comorbidities/short-term complication*, *N* (%)				
Skin	10 (50.0)	16 (100.00)	26 (72.2)	**0.001**
Dyspigmentation	10 (50.0)	16 (100.00)	26 (72.2)	**0.001**
Nail change	0	4 (25.00)	4 (11.1)	**0.018**
Eye	10 (50.0)	14 (87.5)	24 (66.7)	**0.018**
Conjunctivitis	8 (40.0)	9 (56.3)	17 (47.2)	0.332
Corneal epithelial defects	2 (10.0)	4 (25.0)	6 (16.7)	0.230
Synechiae/symblepharon	2 (10.0)	4 (25.0)	6 (16.7)	0.248
Pseudomembrane	3 (15.0)	3 (18.8)	6 (16.7)	0.764
Superinfection	3 (15.0)	7 (43.8)	10 (27.8)	0.053
Vaginal adhesion	0	3 (18.8)	3 (8.3)	**0.043**
Pneumonia	1 (5.0)	1 (6.2)	2 (5.6)	0.871
Pancreatitis	0	1 (6.2)	1 (2.8)	0.257
Adrenal insufficiency	0	1 (6.2)	1 (2.8)	0.257

*Long-term sequel*, *N* (%)				
Skin	5 (25.0)	14 (87.5)	19 (52.8)	**0.001**
Eye	4 (20.0)	9 (56.3)	13 (36.1)	**0.042**
GI (transaminitis)	2 (10.0)	6 (37.5)	8 (22.2)	0.124

*Recurrence*, *N* (%)	1 (5.0)	0	1 (2.8)	0.364

*Mortality rate*, *N* (%)	0	0	0	

^*∗*^Significant values are shown in bold.

**Table 4:** Long-term sequel according to diagnosis and treatment.

	Diagnosis			Treatment		
	SJS, *N *= 20	Overlap-TEN, *N *= 16	*P* value^*∗*^	Supportive care, *N *= 8	Systemic treatment, *N *= 28	*P* value^*∗*^
*Long-term skin sequel*, *N* (%)						
Dyspigmentation	4 (20.0)	14 (87.5)	**<0.001**	3 (37.5)	15 (53.6)	0.423
Xerosis	1 (5.0)	0	0.364	1 (12.5)	0	0.058
Nail loss	0	1 (6.2)	0.257	0	1 (3.6)	0.588

*Long-term eye sequel*, *N* (%)						
Dry eye	4 (20.0)	6 (37.5)	0.244	4 (50.0)	6 (21.6)	0.112
Corneal scar	0	4 (25.0)	**0.018**	1 (12.5)	3 (10.7)	0.887
Keratopathy	0	3 (18.8)	**0.043**	2 (25.0)	1 (3.6)	0.053
Subconjunctival fibrosis	0	1 (6.2)	0.257	0	1 (3.6)	0.588

*Long-term GI sequel*, *N *(%)						
Transaminitis						
Less than 3 m	2 (10.0)	5 (31.2)	0.109	2 (25.0)	5 (17.9)	0.653
More than 3 m	0	1 (6.2)	0.257	0	1 (3.6)	0.588

^*∗*^Significant values are shown in bold.

**Table 5:** Treatment outcomes according to supportive care alone versus systemic treatment.

	Supportive care only, *N *= 8	Systemic treatment, *N *= 28	*P* value
*Length of hospital stay* (d) (mean ± SD)	9.9 ± 7.4	12.3 ± 11.4	0.593

*Comorbidities/short-term complication*, *N* (%)			
Skin			
Dyspigmentation	5 (62.5)	21 (75.0)	0.486
Nail change	0	4 (14.3)	0.257
Eye	5 (62.5)	19 (67.9)	0.777
Conjunctivitis	3 (37.5)	14 (50.0)	0.532
Corneal epithelial defects	2 (25.0)	4 (14.3)	0.473
Synechiae/symblepharon	2 (25.0)	4 (14.3)	0.608
Pseudomembrane	1 (12.5)	5 (17.9)	0.720
Superinfection	1 (12.5)	9 (32.1)	0.532
Vaginal adhesion	1 (12.5)	2 (7.1)	0.629
Pneumonia	1 (12.5)	1 (3.6)	0.331
Pancreatitis	0	1 (3.6)	0.588
Adrenal insufficiency	0	1 (3.6)	0.588

*Long-term sequel*, *N* (%)			
Skin	4 (50.0)	15 (53.6)	0.261
Eye	5 (62.5)	8 (28.6)	0.302
GI (transaminitis)	2 (25.0)	6 (21.4)	0.795

*Recurrence*, *N* (%)	0	1 (3.6)	0.588

**Table 6:** Comparison of clinical features and treatment outcomes between children and adults with Stevens-Johnson syndrome and toxic epidermal necrolysis.

	Children	Adult
*Sex*, %		
Male	44.4	42.1–58.3 [6–13]

*Mean age* (yr)	9.2	40.1–56.6 [6–13]

*Underlying disease*, %	58.4	33.3–76.9 [8, 11–14]

*Causative factor*, %		
Drug-related	72.3	52.4–100.0 [7–9, 11–13]
Non-drug-related	27.7	0–47.6 [7–9, 11–13]

*Prodromal symptoms*, %		
Fever	69.4	59.8–94.7 [11, 13, 14]

*Mucosal involvement*, %		
Oral	97.2	38.6–85.4 [7, 8, 11, 13]
Eye	83.3	59.8–64.4 [7, 8, 11, 13]
Genital	72.2	32.9–41.4 [7, 8, 11, 13]
Anus	16.7	n/a
Nose	-	3.6 [11]

*Associated abnormalities*, %		
GI (Liver) abnormalities	50.0	36.4–48.8 [7, 8, 11]
Renal abnormalities	5.6	9.1–17.1 [7, 8, 11]
Lung abnormalities	-	11.4 [8]
Encephalopathy	-	8.0 [7, 8]

*Treatment*, %		
Specific treatment	77.8	46.6–97.7 [7–9, 11–13]
Supportive care only	22.2	2.3–53.4 [7–9, 11–13]

*Duration of hospital stay* (d)	11.8	10.0–37.0 [6–9, 11–14]

*Short-term complication*, %		
Skin		
Dyspigmentation	72.2	13.7–69.0 [6, 12]
Nail change/loss	11.1	2.9–46.0 [6, 7, 12]
Eye	66.7	0–69.2 [7, 11, 12]
Conjunctivitis	47.2	n/a
Corneal epithelial defects	16.7	Unidentified [11]
Synechiae/symblepharon	16.7	Unidentified [11]
Pseudomembrane	16.7	4.8 [11]
Superinfection	27.8	8.0–56.2 [7, 8, 14]
Vaginal adhesion	8.3	7.7 [12]
Pneumonia	5.6	9.2 [7]
Pancreatitis	2.8	Cases report [15–17]
Adrenal insufficiency	2.8	Case report [18]

*Long-term sequel*, %		
Skin		
Dyspigmentation	50.0	13.7–69.0 [6, 12]
Xerosis	2.8	n/a
Nail loss	2.8	1.1 [7]
Eye	36.1	9.8–77.0 [6, 11, 12, 19]
Dry eye	27.8	31.0–32.4 [6, 12]
Corneal scar	11.1	4.9–15.4 [6, 12]
Keratopathy	8.3	n/a
Subconjunctival fibrosis	2.8	n/a

*Mortality rate*, %	0	6.8–34.4 [6–9, 11–14]

n/a, not available.
